# Design and evaluation of new user control devices for improved ergonomics in flexible robotic endoscopy

**DOI:** 10.3389/frobt.2025.1559574

**Published:** 2025-03-24

**Authors:** Leander Heisterberg, Luigi Manfredi, Dörte Wichmann, Thomas Maier, Peter P. Pott

**Affiliations:** ^1^ Institute of Medical Device Technology, University of Stuttgart, Stuttgart, Germany; ^2^ Division of Imaging Science and Technology, Centre of Medical Engineering and Technology (CMET), School of Medicine, University of Dundee, Dundee, United Kingdom; ^3^ Central Endoscopic Unit of the University Hospital Tübingen, Tübingen, Germany; ^4^ Institute for Engineering Design and Industrial Design, University of Stuttgart, Stuttgart, Germany

**Keywords:** robotic input device, robotic endoscopy, ergonomic, colonoscopy, user control, interface, colonoscopy simulator

## Abstract

**Background:**

The ergonomics of flexible endoscopes require improvement as the current design carries a high risk of musculoskeletal injury for endoscopists. Robotic systems offer a solution by separating the endoscope from the control handle, allowing a focus on ergonomics and usability. Despite the increasing interest in this field, little attention has been paid towards developing ergonomic human input devices. This study addresses two key questions: How can handheld control devices for flexible robotic endoscopy be designed to prioritize ergonomics and usability? And, how effective are these new devices in a simulated clinical environment?

**Methods:**

Addressing this gap, the study proposes two handheld input device models for controlling a flexible endoscope in four degrees of freedom (DOFs) and an endoscopic instrument in three DOFs. A two-stage evaluation was conducted with six endoscopists evaluating the physical ergonomics and a final clinical user evaluation with seven endoscopists using a virtual colonoscopy simulator with proportional velocity and position mapping.

**Results and discussion:**

Both models demonstrated clinical suitability, with the first model scoring 4.8 and the second model scoring 5.2 out of 6 in the final evaluation. In sum, the study presents two designs of ergonomic control devices for robotic colonoscopy, which have the potential to reduce endoscopy-related injuries. Furthermore, the proposed colonoscopy simulator is useful to evaluate the benefits of different mapping modes. This could help to optimize the design and control mechanism of future control devices.

## 1 Introduction

Flexible endoscopes are widely used in colonoscopy procedures, but the way they are controlled can be ergonomically challenging (Shergill and McQuaid, 2019; Otero-González et al., 2022). The design has remained unchanged for decades (Manfredi, 2021). In daily endoscopy routines, complex movements require the simultaneous manipulation of dials while torquing, pushing, or pulling the endoscope (Miller et al., 2022; Shiang et al., 2023). The resulting postures of endoscopists are sometimes referred to as the “endoscopic dance” (Wickramaarachchi and Amarasooriya, 2020). They lead to a high prevalence of endoscopy-related musculoskeletal injuries (Bessone and Adamsen, 2022; Villa et al., 2019; Hansel et al., 2009; Shah et al., 2022; Liberman et al., 2005).

Upcoming robotic systems for flexible endoscopy may offer a solution (Manfredi, 2021; Manfredi et al., 2019; Da et al., 2020). By separating the user interface from the robotic actuator, the design is less constrained and ergonomics can be prioritized. The endoscope is mounted on a robot positioned by the patient’s side, while the user control is with the endoscopist (Sekhon Inderjit Singh et al., 2021). For example, the Bowden cables for control no longer need to be operated by hand, which is often unergonomic and difficult to learn. Instead, they can be remotely controlled *via* a modern interface that does not require any physical strength (Dupont et al., 2021). This new approach to flexible endoscopy control offers a paradigm shift in thinking: The effectiveness of the procedure now heavily relies on the design and usability of the user control. If the control does not meet the endoscopist’s clinical needs, it may compromise the overall acceptance and efficiency of the robotic system. Therefore, it is critical to consider the design and ergonomics of the user control when developing and implementing robotic solutions for flexible endoscopy.

Looking at current robotic endoscopy systems (Boškoski et al., 2021), there are two major challenges hindering progress in this field: the significant size of existing controls or their limited robotic control capacity.

Most current robotic endoscopy user controls have large, complex control mechanisms (Wee et al., 2020; Da et al., 2020), which often take up considerable space in the endoscopy room. Conversely, some systems compromise on functionality by offering limited robotic control. For example, current robotic endoscopy systems such as the Ion Endoluminal System (Intuitive Surgical, Sunnyvale, CA, United States) and the Galaxy System (Noah Medical, San Carlos, CA, United States) focus primarily on bronchoscopic navigation using compact, wheel-based or joystick-like control interfaces (Prado et al., 2024; Bhadra et al., 2024). However, these systems remain limited to bronchoscopic applications and typically lack the DOFs required for advanced gastrointestinal endoscopic procedures, particularly with respect to simultaneous control of multiple instruments or independent rotation and tip manipulation (Ciuti et al., 2020; Da et al., 2020). Non-robotic gastrointestinal endoscopic procedures use four DOFs for basic navigation: insertion/retraction, tip deflection up/down, tip deflection left/right, and rotation and typically 3 DOFs for an instrument: insertion/retraction, rotation, and an action such as opening/closing of a forceps (Lee et al., 2015).

This leaves a gap for ergonomic, compact, handheld interfaces that effectively balance high functionality - such as simultaneous instrument and endoscope control - with ease of use and clinical practicality.

The key questions of this study were:

a) define the requirements for ergonomic user controls,
b) develop concept designs that address the identified needs, and
c) to test these concepts within a clinical setting to assess their feasibility and performance.

## 2 Material and methods

**FIGURE 1:**
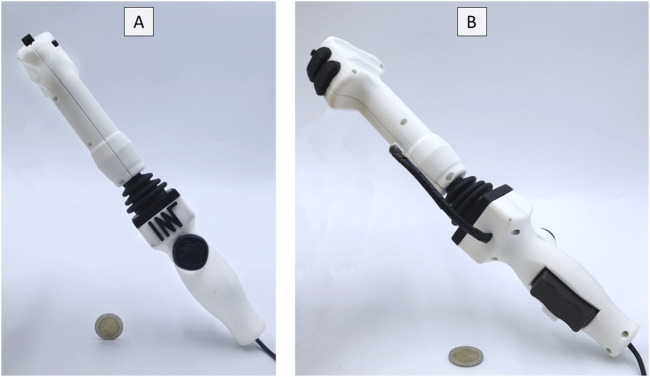
User Control Alpha. **(A)** Front view. **(B)** Rear view.

**FIGURE 2:**
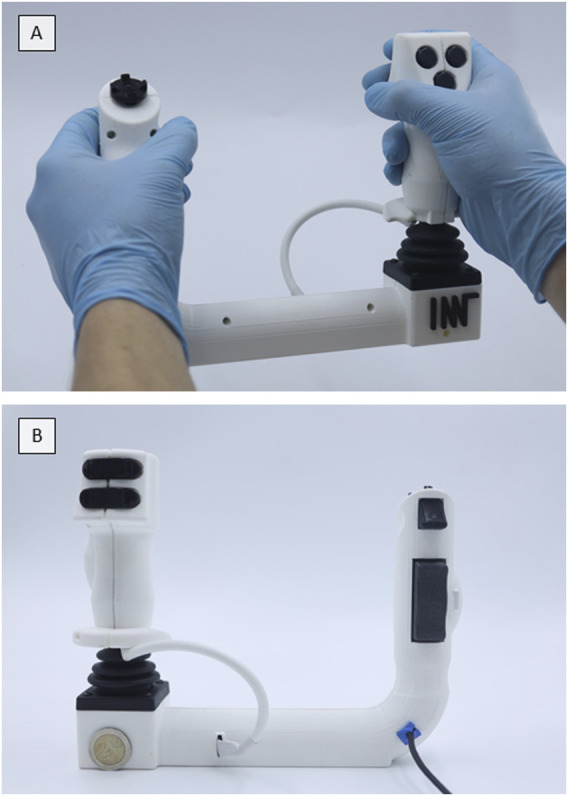
User Control Bravo. **(A)** Front view. **(B)** Rear view.

The design process was based on the German guidelines VDI 2221 (Verein Deutscher Ingenieure, 2019) and VDI 2424 (Verein Deutscher Ingenieure, 2023). Firstly, an extensive literature review, a workflow assessment at the endoscopy department (University Hospital Tübingen, Germany), and a gap analysis was conducted to classify current solutions and areas for improvement. Secondly, as a result of a brainstorming session, six different concept designs were drafted, modelled in clay (Marsclay Medium 8432M, STAEDTLER Industrieplastilin GmbH, Neumark, Germany). Ergonomic evaluation of these clay models was performed by a cohort of 14 medical engineering students and 6 endoscopists, each of whom was asked to rank their top three concepts. This process led to the generation of an overall preference ranking for the six designs. Thirdly, the two most preferred concepts, named Alpha (see Figure 1) and Bravo (see Figure 2), were further refined and buttons and interface elements were integrated. The housings were 3D printed using a Prusa MK3S FDM printer (Prusa Research a. s., Prague, Czech Republic) and polylactic acid filament (PLA). An Arduino Micro board (Arduino S. r.l., Monza, Italy) was used to read and process signals from the interface elements. It has a built-in USB communication, which is recognized by the computer as a human input device and enables a plug-and-play system.

In order to evaluate the usability and ergonomics of the controls, a colonoscopy simulator based on the work of Zhang et al. (2021) was reprogrammed to be used in conjunction with the new user controls. The simulator was developed with the programming environment Unity (Unity Technologies, San Francisco, CA, US). Two mapping modes were implemented, specifically the proportional velocity mode and the proportional position mode. Proportional velocity is a control strategy, in which the velocity of the tip of the endoscope is directly proportional to the displacement of the joystick from its neutral position. In contrast, proportional position is a control strategy, in which the entire deflection of the tip is proportionally mapped to the joystick’s range of motion. These modes are fully configurable during simulation, allowing for adjustments such as setting the maximum velocity or the maximum deflection angle in the proportional position mode.

**FIGURE 3:**
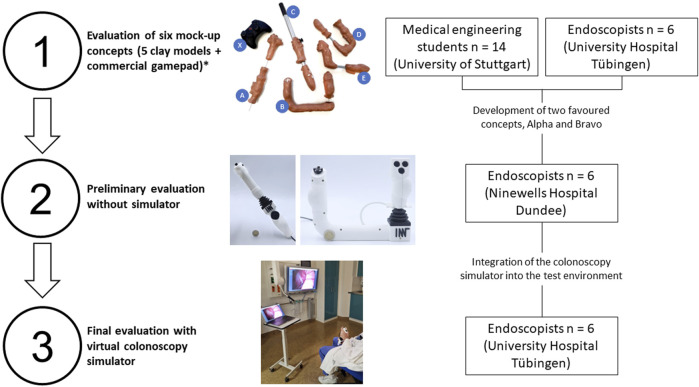
A visual breakdown of the three-stage evaluation process. *Detailed results of clay model evaluation not shown.

Finally, two prospective questionnaire-based clinical user evaluations were conducted. Informed consent was obtained from all participants after they were fully briefed on the study details. Due to the nature of this study, testing prototypes in a simulator, Institutional Review Board approval was not required. The stages of evaluation, from the initial mock-up clay models to the final testing with the colonoscopy simulator, are summarized in Figure 3.

A preliminary evaluation of the two concepts with six gastroenterologists took place at Ninewells Hospital in Dundee, UK, to assess the ergonomics of the first prototypes without the colonoscopy simulator.

Participants included:

• Four consultant gastroenterologists with an average of 18 years of experience in endoscopy (range: 9–30 years), performing an average of 20 endoscopies per week.
• Two trainees with an average of 5 years of experience (range: 3–7 years), performing an average of 12 endoscopies per week.

The study design for the preliminary evaluation at Ninewells Hospital, Dundee, UK, involved randomly assigning participants to either concept Alpha or concept Bravo. Participants were given a brief introduction to the proposed functionality of the control devices, particularly focusing on the button arrangement and design. They were then given time to assess and handle the user controls without navigating through the colonoscopy simulator, basing their judgement solely on the physical design and ergonomic features. Participants rated ergonomics and usability using a six-point Likert scale (1 = “strongly disagree” to 6 = “strongly agree”), which was intentionally designed without a neutral middle point to encourage decisive responses. Participants rated the controls on three domains: (1) ergonomic comfort, (2) suitability for daily clinical routine, and (3) button arrangement. Each domain was rated independently, and responses were analysed to assess overall user preference. Qualitative responses were recorded and analysed thematically to identify common usability concerns, design preferences, and suggested improvements.

**FIGURE 4:**
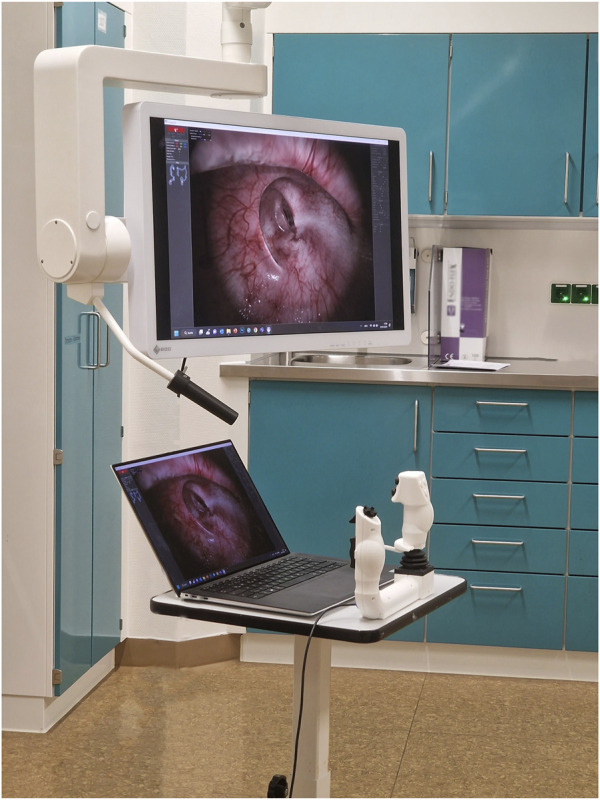
The user controls were evaluated in a clinical setting using the colonoscopy simulator. Here: Model Bravo.

Lastly, the two final prototype models were evaluated with the colonoscopy simulator by seven endoscopists at the Interdisciplinary Endoscopy Unit, University Hospital Tübingen, Germany (see Figure 4).

Participants included:

• Four consultant gastroenterologists with an average of 15 years of experience in endoscopy (range: 7–27 years), performing an average of 30 endoscopies per week.
• Three trainees with an average of 2 years of experience (range: 0.5–6 years), performing an average of 13 endoscopies per week.

In contrast to the preliminary evaluation, the study design for the subsequent evaluation of the user controls at the University Hospital Tübingen involved the use of the colonoscopy simulator. Participants were first randomly assigned to either model Alpha or model Bravo. After a brief introduction to the functionality of the controls and buttons, they were given time to test the user controls by navigating through the colonoscopy simulator. They started with the proportional velocity mode and later tried the proportional position mode. After testing, they completed a questionnaire similar to the one used in the preliminary evaluation, with an additional question to assess the preferred mapping mode.

## 3 Results

As a result of the workflow analysis, key requirements were identified: The user controls should be portable and have four DOFs for endoscope movements (1. Insertion/retraction, 2. Endoscope tip up/down, 3. Endoscope tip left/right, and rotation) and three DOFs for an instrument (1. Insertion/retraction, 2. Rotation, 3. open/close), reflecting the requirements for the majority of colonoscopies. In addition, multiple movements such as insertion, rotation and tip deflection must be performed simultaneously. Based on endoscopist feedback, five programmable buttons were selected to balance functionality with usability while minimizing cognitive load. This aligns with conventional endoscope controls, where essential functions such as suction, air/water infusion, and image capture are typically managed *via* a limited number of tactile buttons (Lee et al., 2015). Although endoscopists perform various tasks, including insufflation, irrigation, suction, contrast adjustment, video recording, and zoom, only a subset of these functions is controlled *via* buttons on the endoscope. To maintain simplicity and ease of use, five programmable buttons were deemed optimal, allowing clinicians to customize them for their most frequently used functions. These identified requirements formed the basis for the design of the user controls.

Both models are lightweight and handheld, with Alpha being rod-shaped (weight: 383 g; dimensions: 5 × 9 × 35 cm^3^) and Bravo being U-shaped (weight: 447 g; dimensions: 24 × 6 × 19 cm^3^). Both use a large joystick to control the deflection of the endoscope tip and its rotation. Furthermore, they have a rocker switch to control the insertion and retraction of the endoscope. A directional pad (D-pad) and a single axis analogue controller, e.g., for grasping movements, are included to control the endoscopic instrument. Each model has five freely programmable buttons for functions such as suction, insufflation, and video recording.

In the preliminary evaluation conducted prior to the use of the colonoscopy simulator, both concepts, Alpha and Bravo, were evaluated. The two concepts scored relatively close, with Alpha scoring an average of 4.5 ± 0.9 out of 6 across all categories and Bravo scoring an average of 4.1 ± 1.6 out of 6. As the overall preference between the two concepts remained split, both concepts were fully developed and tested with the colonoscopy simulator in an advanced testing environment in the final evaluation.

The final clinical user evaluations conducted using the colonoscopy simulator showed that both Alpha and Bravo scored in the top quartile in all categories, namely, 1) ergonomics, 2) whether participants could imagine using the control in their daily routine, and 3) whether they liked the arrangement of buttons and interface elements. However, model Bravo scored slightly higher, with an average score of 5.2 ± 0.7 out of 6 for all three questions. Model Alpha, on the other hand, received an average score of 4.8 ± 1.2 out of 6 across all questions. While there is a slight numerical advantage in favour of model Bravo, statistical analysis identified this difference as a trend (p = 0.07) rather than a statistically significant difference.

**FIGURE 5:**
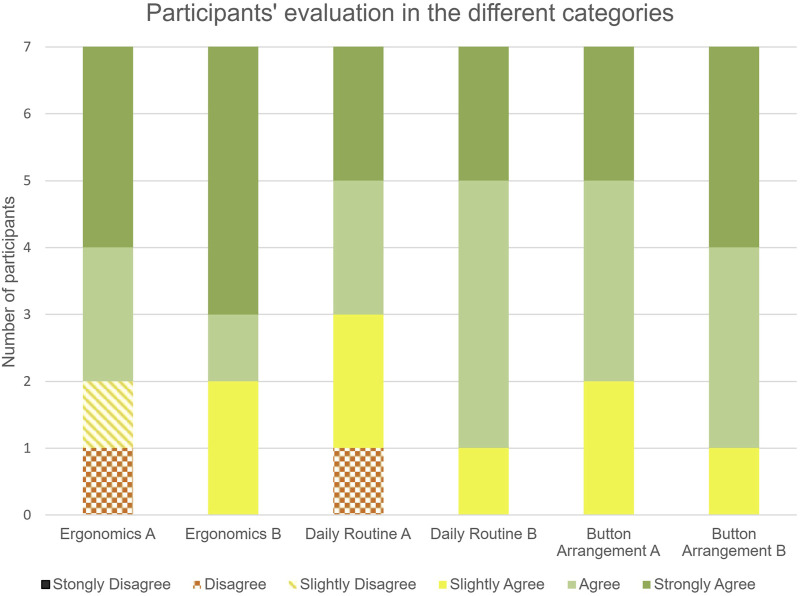
Results of the user rating of models Alpha and Bravo.

Looking at the individual categories for model Alpha, the majority of participants rather agree that the ergonomics are good, that it is suitable for daily routine and that the button arrangement is good (see Figure 5). However, one participant disagrees with the ergonomics and daily routine, and one participant slightly disagrees with the ergonomics.

Regarding model Bravo, it was generally agreed upon by all participants that the ergonomics were good, that it would be appropriate for daily routine, and the button arrangement was satisfactory (see Figure 5). Notably, the response ‘Strongly agree’ was the most selected option across all questions.

**FIGURE 6:**
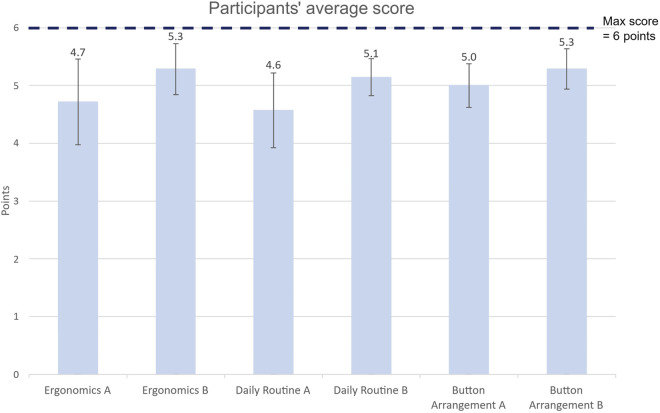
Average score and standard deviation for each category.

When the responses were assigned numerical values in line with the Likert scale, the quantitative analysis confirmed the overall high performance of both models (see Figure 6).

In the context of the mapping mode, participants showed preferences, with four preferring the proportional velocity mode, two preferring the proportional position mode, and one participant indicating no preference.

## 4 Discussion

Both final models were found to be suitable in a clinical setting and, together with the colonoscopy simulator, a realistic testing environment could be created. Significantly, these models replace the traditional steering wheels that are integral to the current design of endoscopic control handles with an ergonomic joystick. This design update allows for simultaneous navigation in all four DOFs of an endoscope. Together with the ability to control an endoscopic instrument within three DOFs, the models offer improved control and minimise the need for an additional endoscopy assistant.

The virtual colonoscopy simulator proved to be a valuable tool in the evaluation of the control devices, providing a far more engaging and realistic experience than in the preliminary evaluations, which only involved physically handling the control and pressing buttons. The simulator’s movements and actions approximate those experienced during real-life diagnostic colonoscopy procedures, providing an authentic environment for testing.

Notably, in the final evaluation phase using the simulator, both models showed improved scores compared to the preliminary assessments. The shift in preference from Alpha to Bravo can be attributed to differences in the test conditions. Initially, participants evaluated the devices based solely on ergonomics, with Alpha’s resemblance to traditional endoscope handles likely influencing its higher rating. However, in the simulator-based evaluation, which emphasized functional performance, Bravo demonstrated superior ease of navigation and control, leading to its higher scores. This suggests that ergonomic preference alone does not predict real-world performance, highlighting the importance of functional testing. In addition, the simulator’s hands-on engagement and learning curve encouraged participants to spend more time with the controls, improving familiarity and confidence. The ability of the simulator to enable clinicians to grasp the concept of robotic colonoscopy and its efficacy may lead to increased openness to such novel developments.

The study also developed two novel, fully adjustable mapping modes for the colonoscopy simulator, enabling either proportional velocity or proportional position control. While a slight majority preferred the proportional position mode in the current study, further research is needed to fully evaluate the advantages of each mapping strategy. It could be beneficial to differentiate between diagnostic and interventional endoscopy when considering the mapping modes. For diagnostic endoscopies, where a quick and comprehensive overview is essential, the direct proportional position mode might be advantageous. In contrast, interventional endoscopy requires delicate movements within a more stable field of view. This may induce a preference for proportional velocity mapping. The simulator provides an important basis for future research to explore the benefits of different mapping methods during robotic colonoscopy.

The prototypes developed in this study represent a novel contribution to the current state of the art. To the best of our knowledge, they are the first handheld user controls capable of independently controlling both the endoscope (with four DOFs) and the endoscopic instrument (with three DOFs). This stands in contrast to more complex systems that perform intricate procedures involving multi-articulated instruments, but in the process, require more elaborate user control devices (Boškoski et al., 2021). Table 1 provides an overview of the range of robotic endoscopy systems, each with different user control devices, ranging from large joysticks to complex console designs and handheld devices.

**TABLE 1:** Overview of robotic endoscopy systems, a description of their user control devices and their state of approval.

Name	Type of user control device	Robotically controlled elements	DOFs	State of approval
Aer-O-Scope (Gluck et al., 2016) (GI View Ltd., Ramat Gan, Israel)	Large joystick mounted on the colonoscopy workstation	Control of the endoscope tip Up/down Left/right	2 DOFs	FDA, CE
ColubrisMX ELS (Atallah et al., 2021) (EndoQuest Robotics, Houston, TX, United States of America)	Open console design: Two omega.7 parallel kinematic devices (Force Dimensions, Nyon, Switzerland); foot paddles to switch between instruments and overtube/videoscope	Two multi-articulating instruments Overtube (Colubriscope) Videoscope	2 × 7 DOFs instruments 2 × 4 DOFs endoscopes → 22 DOFs	IDE, clinical trials ongoing
EndoMaster EASE System (Phee et al., 2012) (EndoMaster Pte Ltd., Singapore)	Two table-mounted arm interfaces to control two endoscopic instruments Endoscope manually controlled by second endoscopist	Two instruments (grasper + cauterizing hook)	2 × 5 DOFs instruments → 10 DOFs	N/A
ETRS – Endoscopic Therapeutic Robot System (Kume et al., 2019) (Kitakyusyu, Japan)	Endoscopic instruments: Two Geomagic Touch™ haptic devices (3D Systems, Inc., Rock Hill, SC, United States of America); endoscope manipulation system with a table-mounted operator control with 60 cm linear track, rotatable handle and mini-joystick; additional linear track for needle injection	Endoscope Two instruments (grasper + knife) Needle catheter	4 DOFs endoscope 2 × 4 DOFs instruments 1 DOF needle → 13 DOFs	N/A
Flex Robotic System (Sethi et al., 2020) (Medrobotics Corporation, Raynham, MS, United States of America)	Omega.3 parallel kinematic device (Force Dimensions, Nyon, Switzerland) Instruments manually operated	Endoscope Up/down Left/right Insertion/retraction	3 DOFs	FDA, CE
Galaxy System (Prado et al., 2024; Bhadra et al., 2024) (Noah Medical, San Carlos, California, United States of America)	Gamepad-like user control with two mini-joysticks and several buttons	Endoscope: Insertion/retraction Up/down Left/right	3 DOFs	FDA
Invendoscope (Boškoski et al., 2021; Straulino et al., 2018) (part of Ambu, Ballerup, Denmark)	Joystick-like interface attached to the colonoscope	Control of the endoscope tip Up/down Left/right	2 DOFs	CE, FDA
Ion (Bhadra et al., 2024) (Intuitive Surgical)	Movable control console with two wheel-based controllers (trackballs)	Endoscope: Insertion/retraction Up/down Left/right	3 DOFs	FDA
K-Flex (Hwang and Kwon, 2020) (Daejeon, Korea)	Two table-mounted master units with multiple joints and translational base Foot clutch for switching to endoscope navigation	Endoscope Two multi-articulating instruments (2 graspers)	4 DOFs endoscope 2 × 5 DOFs instruments → 14 DOFs	N/A
Kyushu University One-Hand Controller (Iwasa et al., 2018) (Fukuoka, Japan)	One-handed control attached to the endoscopic tower	Endoscope control Up/down Left/right Insertion/retraction Rotation	4 DOFs	N/A
Monarch Platform (Graetzel et al., 2019) (part of Ethicon Inc., Raritan, NJ, United States of America)	Gamepad-like control with dual mini-joysticks + buttons for additional functions	Insertion/retraction Up/down Left/right sheath + scope	3 DOFs sheath + 3 DOFs endoscope → 6 DOFs	FDA
STRAS (Zorn et al., 2018) (Strasbourg, France)	Two hand interfaces mounted on a table to navigate the instruments +2 mini-joysticks for the endoscope	Endoscope (Anubiscope) Two instruments	4 DOFs endoscope 2 × 4 DOFs instruments → 12 DOFs	N/A
Three-Limb Teleoperated Robotic System (Huang et al., 2021) (Singapore, Singapore)	Open console design Two omega.7 parallel kinematic devices; foot interface	Endoscope (foot switch) Two instruments (grasper + hook)	4 DOFs endoscope 5 + 4 DOFs instruments → 13 DOFs	N/A
Yonsei University Robotic Colonoscopy (Woo et al., 2017) (Seoul, Korea)	Two table-mounted controllers 1. Large joystick for angulation 2. Rotatable handle with pump mechanism for insertion/retraction	Endoscope control Up/down Left/right Insertion/retraction Rotation	4 DOFs	N/A
Proposed Study (Alpha, Bravo)	Handheld controllers with rocker switches and joystick-style input + additional buttons	Endoscope control Up/down Left/right Insertion/retraction Rotation Instrument control	4 DOFs endoscope +3 DOFs instrument → 7 DOFs	Prototype – Research Study

Looking at the variety, we see systems such as Aer-O-Scope (Gluck et al., 2016) and Invendoscope (Boškoski et al., 2021; Straulino et al., 2018), which limit their focus to straightforward control of the endoscope’s tip, offering two DOFs. On the other end, models such as the ColubrisMX ELS (EndoQuest Robotics) (Atallah et al., 2021) have significantly increased complexity and capability by offering a total of 22 DOFs, allowing control of multiple elements for more sophisticated manoeuvres. Some robotic endoscopy systems, such as Monarch (Auris Health) and Galaxy (Noah Medical), use game-controller-style interfaces (see Table 1). These interfaces, adapted from consumer gaming technology, offer intuitive and familiar control schemes. However, they prioritise multi-functionality over procedural efficiency. In contrast, the proposed user control device integrates dedicated controls for insertion, rotation and tip articulation, ensuring intuitive operation. While game controllers remain a viable option, a dedicated control design tailored to endoscopic workflows may offer a more efficient solution.

Alongside these, the flexible endoscopy field is also witnessing the rise of autonomous systems that eliminate the need for a control device altogether (Martin et al., 2020). While the progress of these autonomous systems is promising, the application is still marked by considerable challenges and a long path that is yet to be traversed (Chadebecq et al., 2023).

Our control devices offer a solution that strikes a balance between ability and complexity. They enable a heightened degree of control and precision while retaining the practicality of a handheld device. The compact, intuitive designs offer a portable solution that integrates smoothly into clinical settings, avoiding the spatial limitations and costliness associated with larger control systems. Moreover, the robustness of the user controls and simulator setup is noteworthy, as it obviates the need for expensive equipment such as an endoscopy tower or a robot.

It is worth acknowledging that the design process had its share of limitations. Although the brainstorming, on which the development was based, and selection of designs followed a systematic approach, it is clear that this process will not come up with all possible solutions. Furthermore, our reliance on experienced endoscopists for testing could potentially introduce a bias; they may have a predisposition towards designs reminiscent of existing endoscope models. This may be one reason why concept Alpha, which has a handle design similar to current flexible endoscopes, was favoured in the initial preliminary evaluations.

The use of 3D printed models for the evaluation of ergonomics also carried certain limitations. Whereas the general acceptance of the design has been demonstrated, refinement of the design that takes into account different hand sizes and optimal positioning of interface elements is required.

The controllers developed in this study are equipped with interface elements designed for manoeuvring endoscopic instruments and include additional buttons that could support functions such as insufflation, suction or irrigation. However, these functionalities remain untested within our simulator framework as the necessary enhancements and modifications have not yet been incorporated into the colonoscopy simulator.

Future research will focus on several key areas: refining the ergonomic design, incorporating haptic feedback systems, extensive simulator testing of different mapping modes, and bridging the gap between user control and a real flexible endoscopy robot. For a transition to a clinical setting, further validation is necessary to establish the effectiveness of the proposed control devices beyond simulated tasks. Comparative trials against existing robotic control interfaces would further clarify usability advantages and limitations.

## 5 Conclusion

In summary, the two control devices offer valuable insights into potential ergonomic user controls for flexible robotic endoscopy. Moreover, the interactive and engaging colonoscopy simulator, when combined with these controls, demonstrates its effectiveness as a training tool and as a means of bringing robotic colonoscopy closer to clinical practice. The study underscores the significance of incorporating ergonomics in the development of robotic endoscopy interfaces and presents a promising avenue for future research in this field.

## Data Availability

The raw data supporting the conclusions of this article will be made available by the authors, without undue reservation.
